# Doxorubicin and vinorelbine act independently via p53 expression and p38 activation respectively in breast cancer cell lines

**DOI:** 10.1038/sj.bjc.6600898

**Published:** 2003-04-15

**Authors:** A A Liem, M V C L Appleyard, M A O'Neill, T R Hupp, M P Chamberlain, A M Thompson

**Affiliations:** 1 Department of Surgery and Molecular Oncology, University of Dundee, Dundee, UK; 2 Department of Molecular and Cellular Pathology, University of Dundee, Dundee, UK; 3 Biomedical Research Centre, University of Dundee, Dundee, UK

**Keywords:** doxorubicin, vinorelbine, signal transduction, p53, additivism

## Abstract

In the treatment of breast cancer, combination chemotherapy is used to overcome drug resistance. Combining doxorubicin and vinorelbine in the treatment of patients with metastatic breast cancer has shown high response rates; even single-agent vinorelbine in patients previously exposed to anthracyclines results in significant remission. Alterations in protein kinase-mediated signal transduction and p53 mutations may play a role in drug resistance with cross-talk between signal transduction and p53 pathways. The aim of this study was to establish the effects of doxorubicin and vinorelbine, as single agents, in combination, and as sequential treatments, on signal transduction and p53 in the breast cancer cell lines MCF-7 and MDA-MB-468. In both cell lines, increased p38 activity was demonstrated following vinorelbine but not doxorubicin treatment, whether vinorelbine was given prior to or simultaneously with doxorubicin. Mitogen-activated protein kinase (MAPK) activity and p53 expression remained unchanged following vinorelbine treatment. Doxorubicin treatment resulted in increased p53 expression, without changes in MAPK or p38 activity. These findings suggest that the effect of doxorubicin and vinorelbine used in combination may be achieved at least in part through distinct mechanisms. This additivism, where doxorubicin acts via p53 expression and vinorelbine through p38 activation, may contribute to the high clinical response rate when the two drugs are used together in the treatment of breast cancer.

Anthracyclines are one of the most active agents in primary adjuvant and palliative treatment of breast cancer (Kroger *et al*, 1999). In metastatic breast cancer, response rates to single doxorubicin treatment range from 52% in previously untreated patients to 28% in patients previously exposed to an alkylating agent (Perry, 1996; Esteva *et al*, 2001). Vinorelbine single-agent treatment of metastatic breast cancer achieves response rates of 35–45% as first-line and 15–30% as second-line therapy (Fumoleau *et al*, 1995; Burstein *et al*, 2001). Despite prior exposure to anthracycline therapy, patient remission was obtained in up to 47% using vinorelbine as a single agent, and for anthracycline-resistant cancers a response rate of 16% was still achieved (Kroger *et al*, 1999). However, the development of drug resistance in cancer cells, particularly against single agents, results in incomplete responses to chemotherapy (Jensen *et al*, 1999; Coley *et al*, 2000; Stavrovskaya, 2000). Com-bined vinorelbine and doxorubicin treatment for metastatic breast cancer has shown higher (74%) overall response rates compared to single-agent treatment (Tsuruo *et al*, 1994; Fumoleau *et al*, 1995; ESMO, 2000), suggesting a synergistic effect of the two drugs.

A detailed knowledge of the mechanisms of action of chemotherapy agents such as doxorubicin and vinorelbine remains incomplete. Two possible routes include signal transduction pathways and the p53 pathway.

Signal transduction is involved in coordinating the cellular response to environmental stresses and is one of the fundamental processes of living cells (Dhanasekaran, 1998; Davis, 2000; Jordan *et al*, 2000; Liem *et al*, 2002). Through these pericellular communications, embryological cells grow, migrate and differentiate, and adult cells maintain their cellular integrity through cell proliferation (cell cycle progression) or cell death (apoptosis) in response to external stimuli (Pawson, 1995; Wang *et al*, 1998; Maemura *et al*, 1999).

Within the mitogen-activated protein kinase (MAPK) family, function of the extracellular signal regulated kinase (ERK, here referred to as MAPK) and p38 appear to be coordinated with JNK in mammary epithelial cells (Agarwal *et al*, 2000; Finlay *et al*, 2000).

p53 is a key gene involved in tumour response to therapy, integrating cellular stress including the action of chemotherapy agents resulting in a range of responses including cell cycle arrest and apoptosis (Hupp *et al*, 2000; Vogelstein *et al*, 2000; Ziyaie *et al*, 2000).

In drug resistance, cross-talk between alterations in signal transduction pathways and the p53 gene has been suggested (Agarwal *et al*, 2001). To identify whether either of these pathways might be relevant in the treatment of breast cancer, this study was performed to establish the effect of doxorubicin and vinorelbine on signal transduction and p53 expression.

## MATERIALS AND METHODS

### Cell culture

MDA-MB-468 and MCF-7 human breast cancer cell lines were obtained from the American Type Culture Collection and cultured in 5% CO_2_ at 37°C using Dulbecco's modified Eagle's medium, supplemented with 10% fetal bovine serum and 1% penicillin/streptomycin. All experiments were performed in triplicate.

### Treatments

MTT cytotoxicity assays (Table 1

**Table 1:** Treatment regimens

	**MCF-7 breast cancer cells**		**MDA-MB 468 breast cancer cells**	
	**Doxorubicin**	**Vinorelbine**	**Doxorubicin**	**Vinorelbine**
IC_50_	10 *μ*M	40 *μ*M	100 nM	30 *μ*M
IC_30_	50 nM	5 nM	—	—

) were performed for doxorubicin and vinorelbine. Using the IC_50_ value for vinorelbine and doxorubicin, MDA-MB-468 and MCF-7 were treated for a total of 4 h. While maintaining the vinorelbine treatment for 3 h, doxorubicin was added either 1 h before (pretreatment with doxorubicin), 1 h after (pretreatment with vinorelbine), or at the same time (combined treatment). Single-agent controls were set up in parallel with pre- and combined treatment regimes. Mitogen-activated protein kinase and p38 activities were determined by kinase assay for the appropriate substrate. p53 expression was determined by Western blotting following treatment for 4 and 24 h with both drugs using IC_30_ values as single agent or in combination.

### Mitogen-activated protein kinase and p38 immunoprecipitation

Following treatment, cells were washed in ice-cold PBS and harvested by mechanical dislodging using a disposable cell scraper (Sarstedt Inc.) in the presence of cell lysis buffer (NEBS), supplemented with phenylmethylsulfonylfluoride for 5 min. The cell lysates were sonicated on ice (Soniprep 150, Sanyo) at full amplitude for four 5 s bursts and centrifuged at 13 000 r.p.m. for 10 min at 4°C. Protein levels were determined spectrophotometrically within the supernatants and 200 *μ*l cell lysates (containing 200 *μ*g total protein) were incubated overnight at 4°C with 15 *μ*l of immobilised phospho-p44/42 MAPK monoclonal antibody or 20 *μ*l of immobilised phospho-p38 MAPK monoclonal antibody for the MAPK and p38 assay, respectively.

### Protein kinase assays and Western blot analysis

Following incubation, the suspensions were microcentrifuged at 13 000 r.p.m. at 4°C for 10 min and washed in 500 *μ*l of 1 × lysis buffer and 500 *μ*l of 1 × kinase buffer. Pellets were suspended in 50 *μ*l of 1 × kinase buffer supplemented with 200 *μ*M ATP and 2 *μ*g of ELK-1 or ATF-2 fusion protein for the MAPK and p38 assays, respectively. Incubation was performed at 30°C for 30 min at 1250 r.p.m. and reactions were terminated with 25 *μ*l of 3 × SDS sample buffer. Samples were boiled at 100°C for 5 min, centrifuged at 10 000 r.p.m., 30 *μ*l was loaded onto a 4–12% Bis-tris (Novex) gels in MOPS running buffer, transferred to a nitrocellulose membrane (Nupage running buffer) and probed with Phospho-ELK-1 or Phospho-ATF-2 primary antibody (1 : 1000) for MAPK and p38, respectively. Visualisation with LumoGlo chemiluminescent reagent was performed following 1 h incubation in 10 ml blocking buffer supplemented with horseradish peroxidase-conjugated anti-rabbit antibody (1 : 2000).

### p53 assay

Treated cells were harvested in 1 ml PBS, centrifuged and supernatants were resuspended in 250 *μ*l urea lysis buffer for 30 min on ice. Protein concentration was determined and 20 *μ*g protein was separated and transferred as described above, incubated for 1 h in CM 1 primary antibody (1 : 1000) and detected using chemiluminescence.

### Densitometry

Protein activity was quantified by scanning the films using densitometry (Molecular Analyst). The activity was plotted as a function of the fold increase of normalised area of kinase activity.

## RESULTS

**Figure 1:**
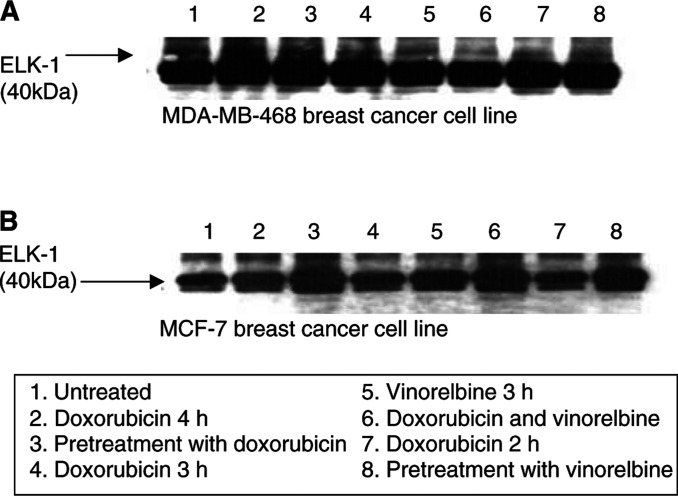
Western blot showing MAPK activity in MDA-MB-468 (**A**) and MCF-7 (**B**) breast cancer cell lines treated with doxorubicin (IC_50_) and vinorelbine (IC_50_) at different time points. Lane 1 shows untreated cells (control); lanes 2 and 3 represent doxorubicin control (4 h) and pretreatment with doxorubicin followed by vinorelbine; Lanes 4 and 5 show doxorubicin and vinorelbine control for the combined doxorubicin and vinorelbine treatment which is shown in lane 6; lanes 7 and 8 show doxorubicin control and vinorelbine pretreatment respectively. The MAPK activity was determined as previously described.

The MDA-MB-468 and MCF-7 breast cancer cell lines showed constitutive MAPK and p38 activation (Figure 1

and Figure 2

**Figure 2:**
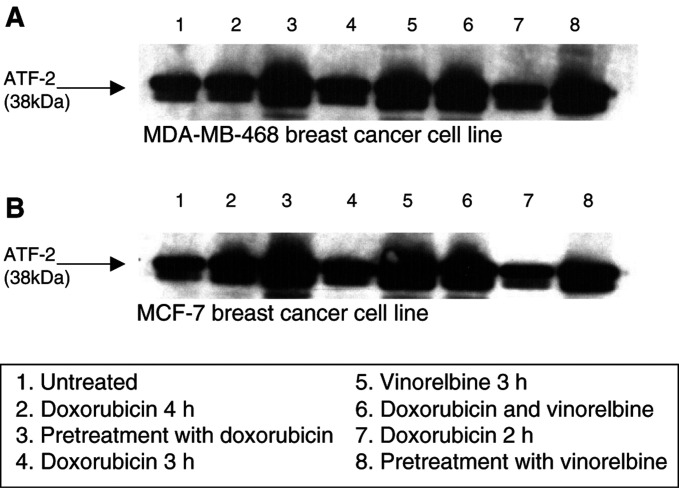
Western blot showing p38 activity in MDA-MB-468 (**A**) and MCF-7 (**B**) breast cancer cell lines treated with doxorubicin (IC_50_) and vinorelbine (IC_50_) at different time points. Lane 1 shows untreated cells (control); lanes 2 and 3 represent doxorubicin control (4 h) and pretreatment with doxorubicin followed by vinorelbine; Lanes 4 and 5 show doxorubicin and vinorelbine control for the combined doxorubicin and vinorelbine treatment which is shown in lane 6; lanes 7 and 8 shows doxorubicin control and vinorelbine pretreatment, respectively. The p38 activity was determined as previously described.

). Doxorubicin treatment did not affect MAPK nor p38 activity and vinorelbine did not significantly affect MAPK activity.

**Figure 3:**
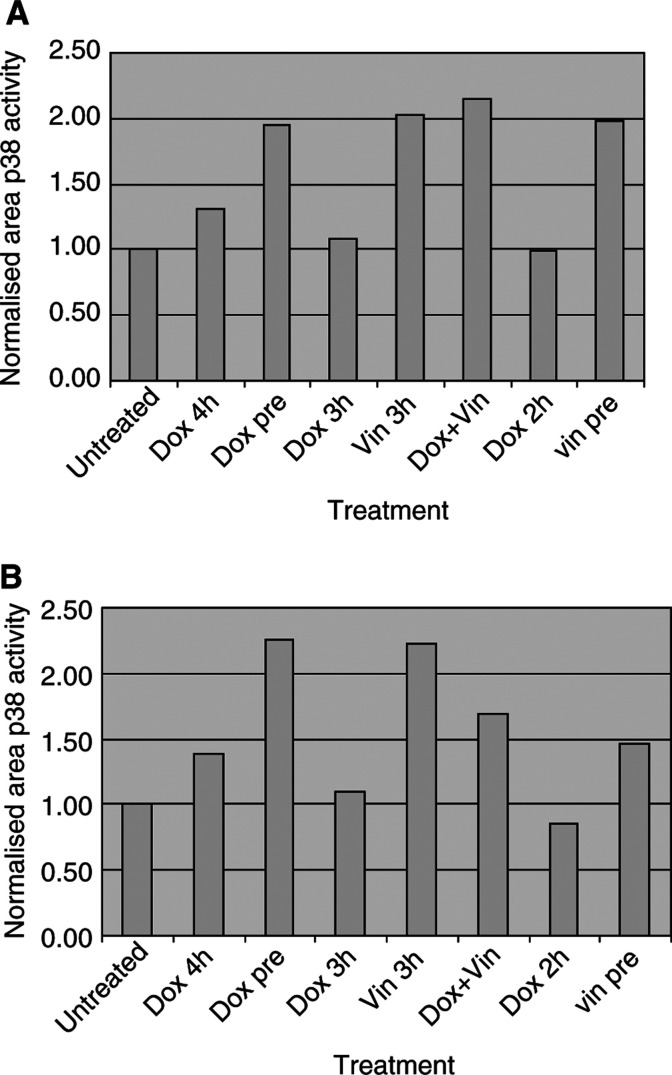
Quantification of p38 activity in MDA-MB-468 (**A**) and MCF-7 (**B**) breast cancer cell lines determined via densitometry following treatment with doxorubicin (IC_50_) and vinorelbine (IC_50_). The *y*-axis represents the fold increase of normalised area of p38 activity. The *x*-axis displays the untreated cells (lane 1); doxorubicin control (4 h) and doxorubicin pre treatment followed by vinorelbine (lanes 2 and 3); the 3 h doxorubicin and vinorelbine treatment (lanes 4 and 5) and combined doxorubicin and vinorelbine treatment (lane 6); doxorubicin (2 h) and vinorelbine pretreatment follwed by doxorubicin is shown in lanes 7 and 8.

However, vinorelbine elicited increased p38 activity (Figure 2, lanes 3, 5, 6, 8). This occurred when vinorelbine was administered as a single agent (Figure 2, lane 5), as pretreatment followed by doxorubicin (Figure 2, lane 8) or given simultaneously with doxorubicin (Figure 2, lane 6). Similar effects were demonstrated when doxorubicin was administered prior to vinorelbine (Figure 2, lane 3). Quantification of p38 activity using densitometry (Figure 3

) showed a two-fold increase of normalised area of p38 activity in vinorelbine-treated MDA-MB-468 and MCF-7 cells compared to the nonvinorelbine-treated cells (Figure 3, lanes 3, 5, 6, 8).

**Figure 4:**
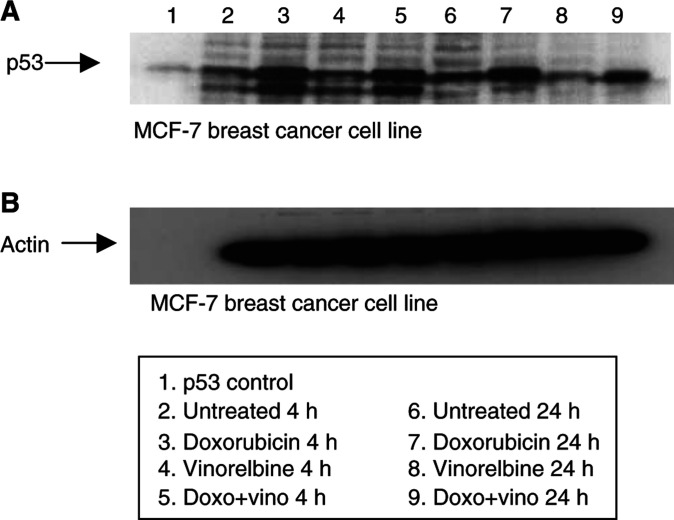
Western blot showing changes in p53 expression in MCF-7 breast cancer cell line following doxorubicin (IC_30_) and vinorelbine (IC_30_) treatment at 4 and 24 h (**A**). A measure of 20 *μ*g protein was loaded. Lane 1 represents p53 control Sf 9 cells. Lanes 2 and 6 show the untreated cells for 4 and 24 h respectively. Single doxorubicin or vinorelbine treatment for 4 and 24 h displayed in lanes 3, 4, 7 and 8, is respectively, and combined treatment for 4 and 24 h shown in lanes 5 and 9. Following treatment, cells were lysed, protein concentration was determined and p53 expression was visualised using Western blotting. Membranes were probed using CM-1 antibody. Probing for actin showed equal loading (**B**).

For p53 expression detected by the CM-1 antibody in the MCF-7 breast cancer cell line (Figure 4

, lanes 3, 5, 7, 9), p53 induction was noted following doxorubicin but not vinorelbine treatment.

## DISCUSSION

This study examined the effect of doxorubicin and vinorelbine *in vitro* on the MAPK family and p53 pathways using two breast cancer cell lines. The two drugs principally act via different mechanisms: doxorubicin intercalates among DNA base pairs resulting in conformational changes in DNA structure and changes in the activity of topoisomerases, whereas vinorelbine is known to disrupt microtubules in the mitotic spindle formation, inducing metaphase arrest during mitosis (Perry, 1996). Constitutive MAPK and p38 activity was confirmed in the MDA-MB-468 and MCF-7 breast cancer cell lines (Sivaraman *et al*, 1997; Hoshino *et al*, 1999). However, when vinorelbine was administered, increased p38 activity was shown in both cell lines. Whether this was because of increased gene expression, increased translation or post-translational modification is unclear. This effect was not seen with doxorubicin, which appeared not to interfere with the p38 activity of vinorelbine. Under the same growth conditions, increased p53 expression, but not enhanced p38 activity, was demonstrated in MCF-7 when treated with doxorubicin, confirming a p53-mediated response to doxorubicin in cells containing a wild-type p53 gene product (Bowcock, 1999; Roses, 1999; Perego *et al*, 2001). While it has been suggested that cross-talk may occur between p38 and p53 (Sanchez-Prieto *et al*, 2000), the current data favour independent activity of p38 and p53 (Bacus *et al*, 2001). These *in vitro* findings provide a molecular basis for the clinical response shown in patients treated with doxorubicin and vinorelbine given in combination. Additivism, where doxorubicin exerts its activity through the p53 pathway and vinorelbine through the MAPK (p38) pathway may account, at least in part, for the high clinical response rate.
